# Genetic structure of a remnant *Acropora cervicornis* population

**DOI:** 10.1038/s41598-021-83112-4

**Published:** 2021-02-10

**Authors:** Steven W. J. Canty, Graeme Fox, Jennifer K. Rowntree, Richard F. Preziosi

**Affiliations:** 1 Working Land and Seascapes, Conservation Commons, Smithsonian Institution, Washington, DC 20013 USA; 2 Smithsonian Marine Station, 701 Seaway Drive, Fort Pierce, FL 34949 USA; 3 Department of Natural Sciences, Ecology and Environment Research Centre, Manchester Metropolitan University, Manchester, M1 5GD UK; 4 Centro de Estudios Marinos, Tegucigalpa, Honduras

**Keywords:** Conservation biology, Ecological genetics, Restoration ecology

## Abstract

Amongst the global decline of coral reefs, hope spots such as Cordelia Bank in Honduras, have been identified. This site contains dense, remnant thickets of the endangered species *Acropora cervicornis*, which local managers and conservation organizations view as a potential source population for coral restoration projects. The aim of this study was to determine the genetic diversity of colonies across three banks within the protected area. We identified low genetic diversity (*F*_ST_ = 0.02) across the three banks, and genetic similarity of colonies ranged from 91.3 to 95.8% between the banks. Clonality rates were approximately 30% across the three banks, however, each genotype identified was unique to each bank. Despite the low genetic diversity, subtle genetic differences within and among banks were demonstrated, and these dense thickets were shown not to be comprised of a single or a few genotypes. The presence of multiple genotypes suggests *A. cervicornis* colonies from these banks could be used to maintain and enhance genetic diversity in restoration projects. Management of hope spots, such as Cordelia Bank, and the incorporation of genetic information into restoration projects to ensure genetic diversity within out-planted populations, will be critical in the ongoing challenge of conserving and preserving coral reefs.

## Introduction

Coral reefs are under severe threat from global climate change. Particular issues include increases in sea surface temperature^1,2^, ocean acidification^3^, and localized stressors such as overfishing^4^ and eutrophication^5^. Coral reefs are reaching a tipping point, with phase shifts from coral to algal dominance becoming increasingly prevalent^6,7^, and potentially irreversible. As the biological and physical structure of coral reefs change, ecosystem service provision and the resilience of these systems to future stresses is reduced^8^. The loss of ecosystem services is of concern for coastal populations who rely on them, both directly, e.g., for fisheries^9^, and indirectly, e.g., for storm protection^10^. To abate phase shifts and conserve coral reef biodiversity, urgent management is required at both global and local scales.

Within the Caribbean, average coral cover declined from 34.8% in 1970 to 16.3% in 2012^11^. Of significance during this period was the loss of approximately 80% of Caribbean Acroporid corals, which was triggered by an outbreak of white band disease in combination with multiple climatic events, including hurricanes^12^. During the intervening decades, there has been little to no recovery of these populations, and both *Acropora palmata* (elkhorn coral) and *A. cervicornis* (staghorn coral) have been listed as critically endangered by the International Union for Conservation of Nature^13,14^. However, remnant Acroporid populations have been documented throughout the Caribbean, e.g. in Mexico and Belize^15^, Honduras^16^, Guadeloupe^17^, U.S. Virgin Islands, St. Vincent and the Grenadines, Bonaire and Curacao^18^.

Low genetic diversity and high clonal frequency can be common within Acroporid populations^17^. Asexual or clonal reproduction strategies are associated with maintaining and preserving existing genetic diversity during periods of population decline and poor recruitment from sexual reproduction, a particular concern in fragmented and remnant populations^19^. Critically, remnant populations have the potential to become sexually extinct after prolonged periods of clonal growth, if recruitment of sexually reproduced individuals from other populations is low^20^. Which may be attributed to the Allee effect, as fertilization success in broadcast spawning corals, such as Acroporids, is density dependent^21^. Caribbean Acroporid populations are generally considered to be dominated by clones, and thus non-sexual reproduction, however, there are exceptions to this; high levels of genetic diversity have been observed in populations of *A. palmata* in Mexico, Belize^15^, and the Eastern Caribbean^22^, and *A. cervicornis* populations along the Florida Reef Tract^23^. Higher levels of genetic diversity suggest a greater prevalence of sexual reproduction, and within the Eastern Caribbean this has been considered to be related to habitat characteristics^22^. Sexual reproduction has the potential to promote genetic diversity and, therefore, the ability to respond to environmental change within a species, increasing resilience in the face of environmental stresses^24^, and may enhance species diversity at the community level^25^. Further, areas with high genetic diversity have been associated with higher coral cover^26^. Within *A. cervicornis* clumping of ramets, or clonal genotypes, has been observed across the reef scape^22,27^. This clumping suggests low genetic diversity at the micro-scale and increased genetic diversity at the macro-scale, therefore greater allelic diversity is observed in larger populations.

Whilst the presence of remnant populations of threatened species is a cause for hope, there is a realization that coral reefs are unlikely to return to past configurations in terms of community assemblage. Therefore, the challenge for both the scientific and management communities is to maintain ecosystem function in these critical systems^28^. There is concern that recovery by natural processes may not be sufficient, e.g., if coral settlement is inhibited by algae^29^, interventions such as anthropogenic restoration may also be required^30^. In light of this, initiatives are focusing on remnant populations as potential seed populations^31^, at least at the local scale.

**Figure 1:**
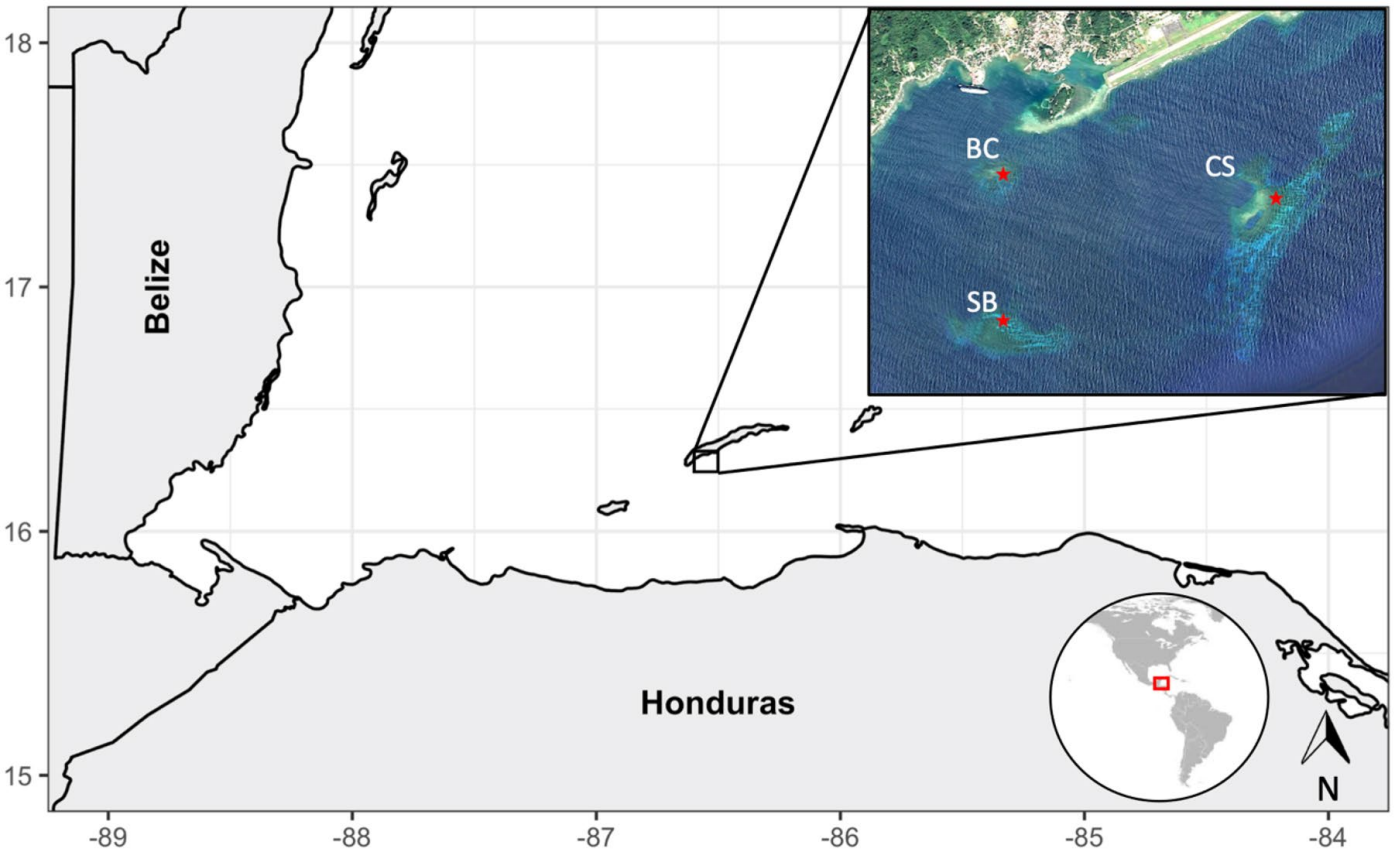
Map of the Honduran north shore, highlighting the location of Cordelia Bank Site of Special Importance to Wildlife, and the three banks with dense thickets of *Acropora cervicornis*, BC—Big Cay, CS—Cordelia Shoal, SB—Smith Bank, approximate sampling locations are indicated by red stars. Maps were created with R Studio version 1.2.1335^37^ using satellite images provided by Google Maps.

Remnant populations of *A. palmata* and *A. cervicornis* have been observed in Honduras^16^, and Guadeloupe^17^ and corals from these populations have the potential to seed the recovery of Caribbean Acroporid populations^31^. The Cordelia Bank Site of Special Importance to Wildlife is one such area. The reef system, located in the Honduran Caribbean, was identified to contain extensive *A. cervicornis* colonies^16^ (Fig. 1). Due to the prevalence of colonies, the area is being considered as the potential source of colonies for use in local restoration projects. Knowledge of the genetic composition of colonies prior to restoration is essential^32–34^, but to date, no genetic studies have been conducted on the colonies within Cordelia Bank Site of Special Importance to Wildlife. It is not known if a single, or multiple genotypes are found within these populations. We used microsatellite markers to assess the genetic diversity of individual sexually mature colonies of *A. cervicornis* across three banks within Cordelia Bank Site of Special Importance to Wildlife. Our aim was to provide a genetic baseline of colonies within the protected area prior to the implementation of restoration projects that plan to use these colonies as a source population.

## Materials and methods

### Study site and sample collection

Cordelia Bank (N 16.30°; W 086.52°) was officially declared a Site of Special Importance for Wildlife in 2012, by the Honduran government^35^. The area consists of four offshore banks, Cordelia Shoal, Smith Bank, Big Cay and Little Cay, located approximately one mile south-west of the island of Roatan, Bay Islands, Honduras (Fig. 1). The area was given protective status due to the abundance of *A. cervicornis*, with colonies estimated to extend over an area of 63,440m^2^, across three primary banks^36^.

Sampling was conducted in April 2014 on three of the four banks: Big Cay; Cordelia Shoal and Smith Bank, based on the presence of high densities of *A. cervicornis,* as identified by Riegl et al.^36^. Sampling was not undertaken on Little Cay due to weather constraints. In-water observations were first conducted to confirm the suitability of sampling areas and ensure that the selected locations had close to 100% *A. cervicornis* coral cover. For each bank, 100 5 m × 5 m sampling cells were initially established across a 50 m × 50 m grid. Due to inclement weather and the risk of causing damage to the reef, the sampling grid was modified on the shallowest banks: Big Cay to 50 m x 25 m and on Cordelia Shoal to 50 m × 30 m. This provided a combined area of 5250m^2^, representing over 8% of the total estimated cover of *A. cervicornis* cover across the three banks.

The sampling grid was laid out on the reef using four 50 m measuring tapes, to demark the sampling area. Three additional measuring tapes were used to make horizontal internal lines at 5 m intervals, to create two adjacent rows. Flagging tape placed at 5 m intervals along the measuring tapes was used to demark individual sampling cells of 5 m x 5 m. Once sampling was completed for these two rows, measuring tapes were moved further up the reef to create two subsequent rows and repeated until the sampling was complete. Sampling started at the deepest part of the reef, working up to the shallows.

Corals were sampled by taking a small cutting, 2–3 cm long, from the branch of a single *A. cervicornis* colony within each of the sampling cells. Cuttings were placed into individually labelled bags containing seawater, taken ashore and then transferred to 100% ethanol and frozen for storage prior to genetic analyses. Sampled colonies were chosen if: (1) they were the dominant colony within the grid that had a basal attachment, and had not been sampled in a previous grid; and (2) they had a minimum branch length of 17 cm, to ensure they were sexually mature^38^. If the dominant colony had been sampled previously, the next largest colony in the grid was sampled instead. Sampling only mature colonies was a specific strategy to detect the full genetic composition of the potential reproductive stock of *A. cervicornis* within the protected area. Each sample was geo-referenced, with GPS coordinates recorded by a snorkeler at the surface, and depth recorded to 0.1 m accuracy using a Matrix dive computer (Mares™, Rapallo, Italy). A total of 205 samples were collected and successfully genotyped from across three offshore banks, Big Cay n = 50, Cordelia Shoal n = 57, Smith bank n = 98 (Table 1).

**Table 1:** Description of ramet and clonal diversity of *Acropora cervicornis* within the Cordelia Bank Site of Special Importance to Wildlife.

	N	N_g_	N_g_/N	C_g_	C	Colonies per ramet			Percentage clones (%)
						Maximum	Minimum	Mean	
Big Cay	50	42	0.84	7	15	3	2	2.1	30.0
Cordelia Shoal	57	44	0.77	4	17	10	2	4.3	29.8
Smith Bank	98	75	0.77	10	33	8	2	3.3	33.7
Combined	205	161	0.79	21	65	10	2	3.1	31.7

N, is the total number of colonies sampled; N_g_, is the number of unique genotypes identified; N_g_/N is the genotype to colony ratio; C_g_ is the number of ramets identified; C is the total number of colonies identified as clones.

No significant difference in the number of clones per bank (chi-squared = 4.125 p = 0.127), the number clonal genets per bank (chi-squared = 1.348 p = 0.510), or the mean ramets per genotype per bank (chi-squared = 0.392 p = 0.822) were observed.

### Genotyping

Fragments of approximately 1 cm length of coral were used for DNA extraction. These were crushed using a 0.5″ chisel and transferred to a microcentrifuge tube, to which Qiagen DNeasy Blood and Tissue ATL buffer and Proteinase K were added. Samples were then placed in an Eppendorf thermomixer (Hamburg, Germany) at 56 °C and 600 rpm for 4 h. Once digestion was completed, DNA extractions followed the Qiagen DNeasy Blood and Tissue protocol. DNA concentration was calculated using a BioTek Epoch Microplate Spectrophotometer (Winooski Vermont, United States), and where necessary, DNA was concentrated to ensure that 20 ng of DNA was used in each subsequent amplification reaction.

Individual *A. cervicornis* colonies were genotyped using fourteen polymorphic microsatellite loci: 0166, 0181, 0182, 0192 & 0207^39^ and 0513, 0585, 1195, 1490, 2637, 5047, 6212, 9253 & 0007^40^. Polymerase chain reactions were carried out on BIO-RAD T100™ Thermal Cyclers (Hercules California, United States), with an initial denaturation step at 95 °C for 5 min followed by 35 cycles of 95 °C for 20 s, 51–55 °C for 20 s, 72 °C for 30 s, and a final extension of 30 min at 72 °C, with the exception of 0007. This marker required an initial denaturation step at 95 °C for 5 min followed by 31 cycles of 95 °C for 15 s, 55 °C for 15 s, 72 °C for 30 s, and a final extension of 30 min at 72 °C. Genotyping was performed using an ABI 3730xl automatic DNA analyzer (Applied Biosystems, Waltham, Massachusetts, United States). An internal size standard (GeneScan 500-LiIZ, Applied Biosystems) was used for accurate sizing. Electropherograms were analyzed using GeneMapper v.5.0 and alleles were subsequently binned with the R-package Msatallele version 1.02^41^. Genotyped colonies with more than 20% missing data (missing data from three or more loci) were removed from subsequent analyses. The locus 0192 did not genotype evenly across samples and therefore was removed from the analysis. All of the laboratory and computer work was conducted in and with the support of the Laboratories of Analytical Biology facilities of the Smithsonian’s National Museum of Natural History (Washington, D.C., United States).

### Data analysis

Clones were identified as genetically identical to another individual, and these individuals were then assigned to a ramet, using a two-step process. Firstly in GenoDive^42^, a distance matrix was calculated using a stepwise mutation model, where missing data was not counted, the threshold was set at zero, and clonal structure was tested using a stepwise mutation model of the corrected Nei’s diversity index statistic with the randomize alleles over individual colonies of all three banks, using 999 permutations. These outputs were cross-checked in GenAlEx 6.5^43^, which allows for the inclusion of colonies with missing data, using the matching function where all data is considered as a single population and alleles are codominant. Through this step, an additional three colonies were identified as clones and assigned to corresponding ramets. Where clones were corroborated, a single representative of the ramet was used in further analysis. Summary data of each locus (number of alleles, expected and observed heterozygosity) were calculated for each population, and pairwise F_ST_ and Nei unbiased genetic identity tests were conducted in GenAlEx 6.5^43^.

Population structure of *A. cervicornis* colonies was analyzed using the software *STRUCTURE*^44^, using an admixture model with allele correlation. The Burn-in period length was set at 100,000, and the number of Markov chain Monte Carlo replications after Burn-in was set at 100,000. We ran the model with K values of 1 through 10, and with 10 permutations for each K value. To identify the optimal K, the model outputs were analyzed in STRUCTURE HARVESTER^45^, with the highest delta K value used to identify the optimal K value. Mantel tests were conducted to test for correlations between genetic distance and geographic distance, and genetic distance and depth, and a partial Mantel test to test for partial correlations among all three, these analyses were conducted using the *vegan* package^46^. Additional Chi-squared analyses of clonal diversity across the three banks were conducted in R Studio version 1.2.1335^37^.

## Results

### Clonal genetic analysis

**Figure 2:**
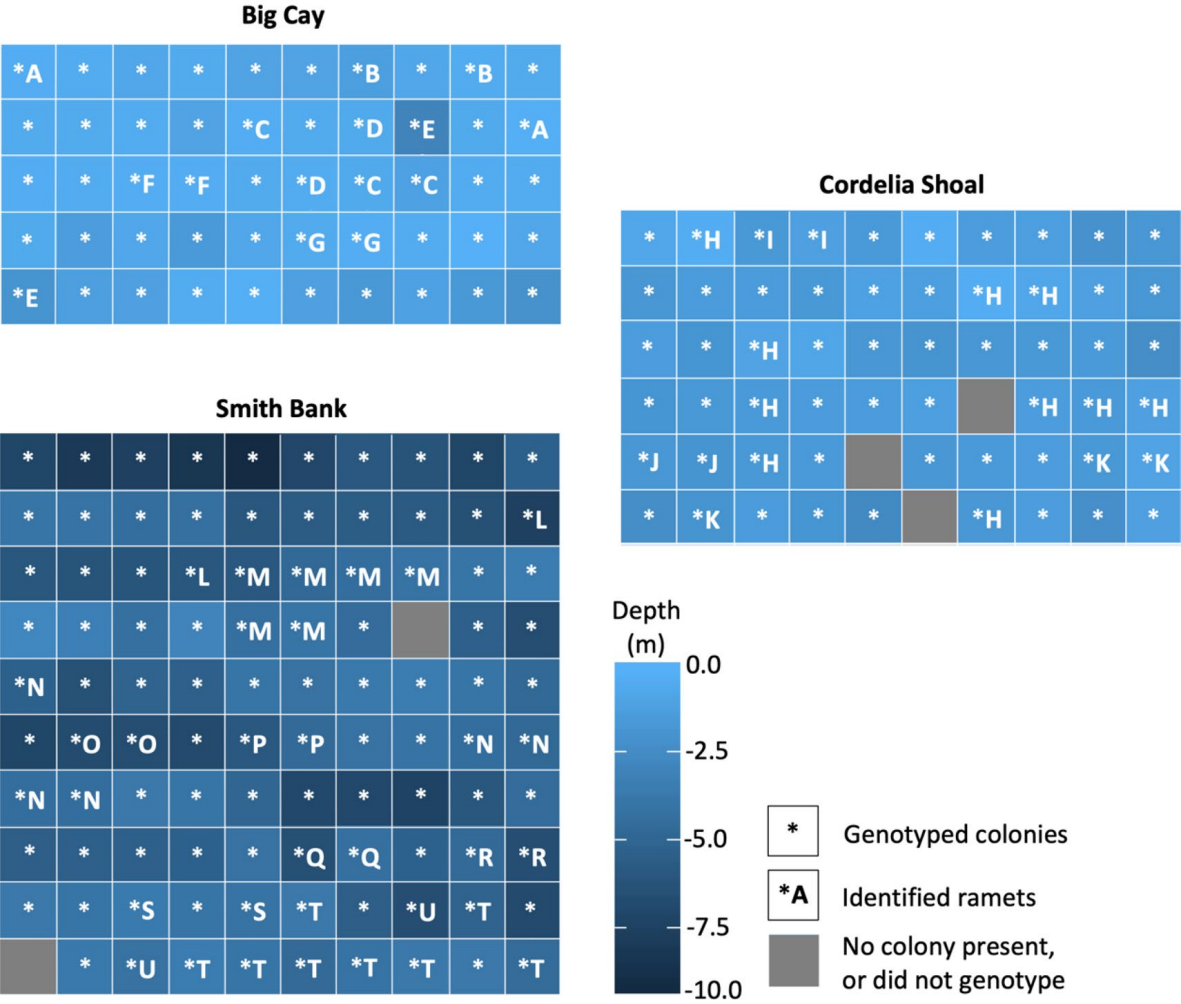
Depth profiles of sampled *Acropora cervicornis* colonies and location of clones within the three banks of Cordelia Bank Site of Special Importance to Wildlife, each letter represents a unique ramet (clonal genotype).

A total of 65 clones, belonging to 21 ramets, were identified across the three banks, and were unique to individual banks (Fig. 2, Table 1). Approximately one third (31.7%) of all colonies sampled were identified as a clone. Ranging from 29.8% to 33.7% across the three banks, no significant differences in the occurrence of colonies identified as clones were observed (chi-squared, *p* = 0.846). Across all alleles, the number of ramets varied among banks, as did the mean number of colonies per ramet, and neither was significant (chi-squared, *p* = 0.654, chi-squared, *p* = 0.132 respectively), nor was there an interaction between the number of ramets and the number of colonies per ramet, per bank (chi-squared, *p* = 0.654) (Table 1).

### Genetic structure

**Figure 3:**
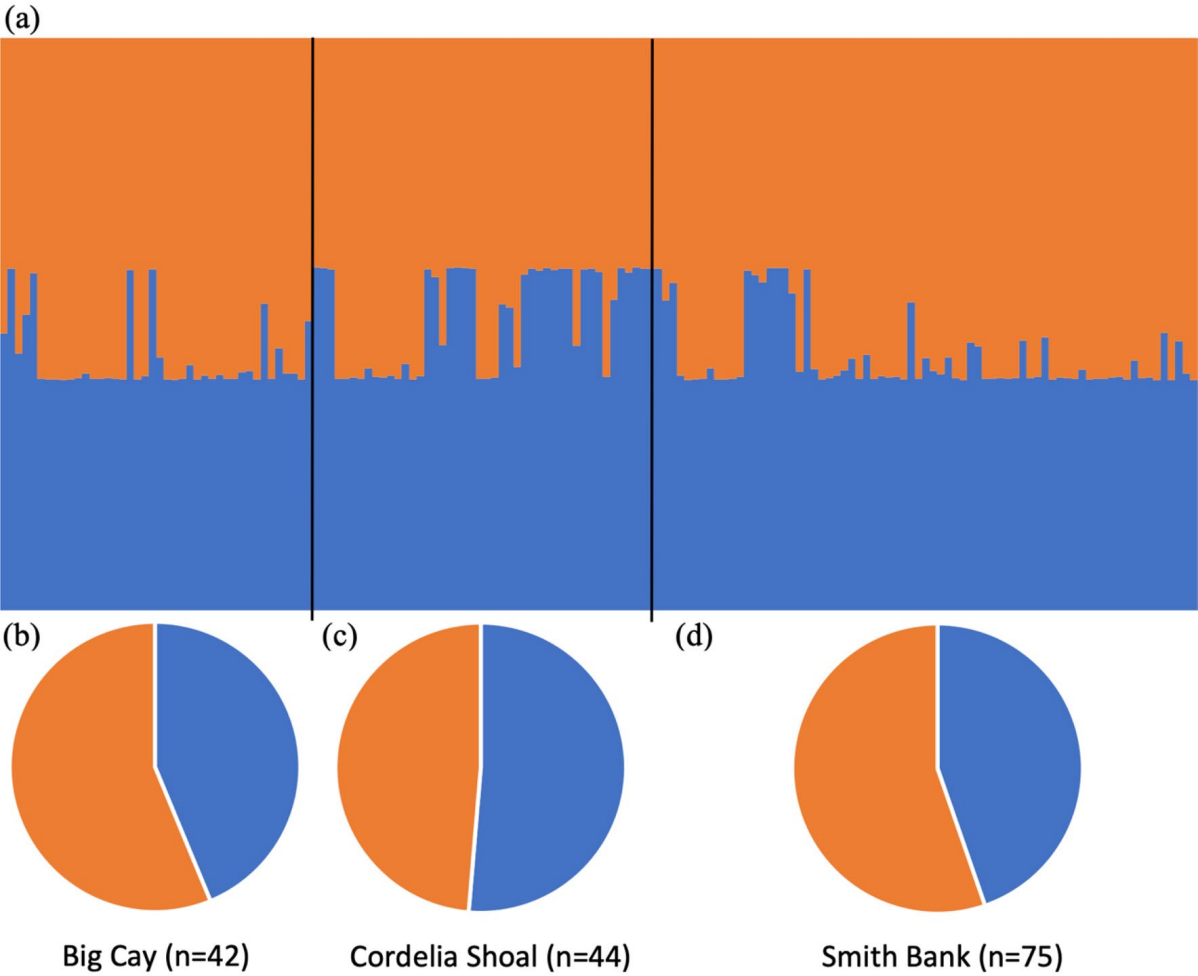
STRUCTURE outputs for all genotypes (K = 2), mean of 10 permutations, for each colony within each of the banks (**a**); and the mean cluster classifications of all colonies within Big Cay (**b**); Cordelia Shoal (**c**); and Smith Bank (**d**). Blue—Cluster 1; and Orange—Cluster 2.

Genetic diversity across the Cordelia Bank Site of Special Importance to Wildlife was low (*F*_ST_ = 0.020), varying from *F*_ST_ = − 0.032 to 0.102 across the individual banks (Table 2). Pairwise *F*_ST_ analyses suggested low genetic differentiation among the colonies sampled across the three banks, with values ranging from 0.014 to 0.025. Nei’s unbiased genetic identity analyses corroborate these findings, indicating limited genetic differentiation among the three banks, ranging from 0.913 to 0.958, with greatest similarities observed between Big Cay and Smith Bank (Table 3). A weak significant relationship was observed between genetic distance and geographic distance (Mantel test, *r* = 0.108, *p* = 0.002), no relationship was observed between genetic distance and depth (Mantel test, *r* = − 0.038, *p* = 0.909) or between genetic distance and a combination of geographic distance and depth (Partial mantel, *r* = − 0.089, *p* = 0.993). Population structure analyses highlight the similarities in the genetic structure of *A. cervicornis* colonies within and across the sampling locations, with individual colonies having both K clusters well represented and no individual colony fully assigned to either cluster (Fig. 3a). However, subtle differences in cluster allocation were observed at the bank level. Greater proportions of cluster 2, 56% and 55%, were presented in colonies on Big Cay (Fig. 3b), and Smith Bank (Fig. 3c), respectively. Whereas colonies on Cordelia Shoal (~ 51%) have a slightly greater proportion of cluster 1 (Fig. 3d).

**Table 2:** Genetic diversity at 13 microsatellite loci for *Acropora cervicornis* for the three sample sites of Cordelia Bank Site of Special Importance to Wildlife.

	Big Cay		Cordelia Shoal		Smith Bank		All sites	
	N_a_	*F*_ST_	N_a_	*F*_ST_	N_a_	*F*_ST_	N_a_	*F*_ST_
0166	7	− 0.086	6	0.017	7	0.021	9	0.024
0181	8	− 0.081	12	0.026	12	0.064	13	0.015
0182	10	− 0.124	11	0.006	14	0.031	16	0.013
0207	8	− 0.094	7	− 0.060	8	0.014	9	0.034
0513	6	− 0.088	8	0.009	8	− 0.190	10	0.009
0585	7	0.020	7	0.146	4	− 0.013	7	0.004
1195	4	0.071	5	0.242	6	0.372	6	0.021
1490	5	0.220	3	0.634	5	0.404	6	0.065
2637	7	0.012	6	− 0.178	10	0.030	10	0.005
5047	7	− 0.159	7	0.293	7	− 0.068	9	0.023
6212	12	− 0.026	10	0.120	13	0.122	15	0.006
9253	2	− 0.024	3	− 0.018	4	− 0.030	6	0.047
0007	10	− 0.060	12	0.093	12	0.063	13	0.017
Overall		− 0.032		0.102		0.063		0.020

Only one representative of each clonal genotype is included in the analysis. N_a_, number of alleles; *F*_ST_, Fixation coefficient.

**Table 3:** Pairwise *F*_ST_ and Nei unbiased genetic identity values of *Acropora cervicornis* colonies from three banks within the Cordelia Bank site of special importance to wildlife.

	Big Cay	Cordelia Shoal	Smith Bank
**Pairwise F**_**ST**_			
Big Cay	–		
Cordelia Shoal	0.025	–	
Smith Bank	0.014	0.017	–
**Nei unbiased genetic identity**			
Big Cay	–		
Cordelia Shoal	0.913	–	
Smith Bank	0.958	0.939	–

## Discussion

The extensive thickets of *Acropora cervicornis* colonies within the Cordelia Bank Site of Special Importance to Wildlife are comprised of numerous genetically distinct colonies, however genetic diversity within and among the three banks was low. Clones were observed in each of the three banks, with mean clonality across the three banks at 31.7%. However, genotypes were unique to individual banks suggesting spatial structuring between the banks, which has been observed in other populations^22,27^. The high number of unique genotypes which was observed (mean N_g_/N = 0.79) differs from how *Acropora* reefs are generally considered and what has been observed in other populations, e.g. Florida (N_g_/N = 0.33), Belize (N_g_/N = 0.39)^47^ and Guadeloupe (N_g_/N = 0.01)^17^. The systematic sampling methodology used in this study, which ensured that multiple sexually mature colonies were sampled, can maximize the genetic diversity observed. This may have contributed to the lower prevalence of clonality than that observed in other studies. However, similar to this study, high frequencies of unique genotypes and low clonality have been observed in *A. cervicornis* populations, e.g., the Bahamas (N_g_/N = 0.64), Turks and Caicos (N_g_/N = 0.65), and Panama (N_g_/N = 0.66). The high frequency of distinct, but similar, genotypes within and across the three banks of Cordelia Bank Site of Special Importance to Wildlife provide a small, but potentially significant, reservoir of genetic diversity. Whilst genetic diversity may be low within, and across *A. cervicornis* populations, significant, but weak, genetic differences driven by geographic distance were observed. This research did not address the drivers of genetic differentiation, and therefore we can only postulate that the genetic differentiation observed is a result of natural selection or a founder effect. These subtle genetic differentiations could be key in allowing individual colonies to adapt to future stressors, and therefore it is critical that this diversity is protected and conserved, this will become more important if these reefs, and others, are not restored through sexual recruitment.

Maintaining this genetic diversity will be crucial if sexual reproduction events are triggered in the future; such events provide the opportunity to increase genetic diversity within populations^48^. Spawning activity within Acroporid populations has been observed in the Florida Keys, Panama and Belize, July through October^49^, and spawning in Belizean *A. palmata* has been observed most years from 2010–2019 (Personal communication, M. Scott Jones, Smithsonian Marine Station). Monitoring for spawning activity of *A. cervicornis* in Cordelia Bank Site of Special Importance to Wildlife was last conducted during the full moons of June, July and August 2013. No spawning was observed in *A. cervicornis* colonies during this period, however, spawning of *Orbicella annularis* and *O. faveolata* was observed during the August full moon (Personal observations, SWJC). Spawning in multiple *A. palmata* colonies in Tela Bay, Honduras, was observed during the same August 2013 full moon (Personal communications, Andrea Rivera, Universidad Nacional Autónoma de México). These observations suggest that environmental cues to trigger spawning are present in the region. Whilst the potential for natural recovery exists, even where spawning has regularly been observed, the overall cover of Acroporids has remained low^50^. It is therefore likely that further intervention is required to assist in the recovery of Caribbean Acroporid populations.

Restoration is becoming an increasingly popular tool for conservation and management of marine habitats^51,52^ and within the Caribbean over 150 projects in more than 20 countries have been implemented^53^. Coral gardening, a preferred technique in the Caribbean, inherently limits genetic diversity as the technique focuses on the growing and out-planting of clones^54^. Despite genetics being an important factor that complements traditional restoration ecology methodologies^55^, and ensures ecological and evolutionary processes are incorporated into the restoration process^56^. Genetic diversity provides colonies with the potential to respond to changing environmental conditions, and where no genetic variation exists, responses are limited to phenotypic plasticity to deal with these stressors. During restoration there is the potential for the loss or reduction of fitness in the restored population, driven by founder effects, genetic swamping and inbreeding or outbreeding depression^32^. Greater attention needs to be given to genetic diversity when restoring systems^57^, especially when projects are dominated by a single species, such as coral gardening of *A. cervicornis*, the genetic diversity represents the primary biodiversity of the habitat. Genetic composition of out-planted colonies is one of many important criteria that should be considered within a best practices approach to restoration^58^.

Understanding the drivers of existing genetic structure and environmental conditions will be important in the successful management and conservation of these populations, and of restoration projects that use colonies from these populations. If a restoration project using colonies from Cordelia Bank Site of Special Importance to Wildlife is to be implemented, then the genetic diversity across the banks observed in this study should be considered. Selectivity of colonies during the restoration process can ensure representation of a range of genotypes maximizing the potential for evolutionary adaptation of corals within a restored area. There is an important caveat that underlies this potential and the future of the corals within Cordelia Bank, the Caribbean, and globally. Understanding and reversing the ultimate localized drivers of reef decline (e.g., overfishing and eutrophication) must be part of comprehensive local and regional management strategies. The coral populations of the Mesoamerican barrier reef system, which encompasses ﻿Cordelia Bank, are under pressure from ocean acidification, hurricanes, pollution and fishing, and at high risk from mass bleaching over the next decades, and the ecosystem has been categorized as critically endangered by the IUCN^59^. In the specific case of Cordelia Bank, fishing and recreational activities have been excluded from highly sensitive areas, however, urban runoff and untreated effluents from Coxen Hole, and the proximity of two major cruise ship docks and an international airport, represent potential major threats^60^. If coral reefs are to have sufficient resilience to climate change and continue to provide critical ecosystem services to the coastal communities that depend on these resources, the drivers of their decline must be reduced. Whilst management cannot prevent the damaging effects of major disturbances, it can provide protection to reefs that have the greatest potential to be resilient and contribute to recovery through natural processes^61^. Natural regeneration promotes more complex and resilient systems than active restoration^62^, therefore restoration should be considered as one of a multitude of management tools in the conservation of coral reefs.
